# Editorial: Research advances on forest tree functional genomics and breeding

**DOI:** 10.3389/fpls.2024.1508507

**Published:** 2024-12-04

**Authors:** Juan Du, Tianqi Ye, Yi An, Yicun Chen, Jack Wang, Jiehua Wang, Mengzhu Lu, Quanzi Li

**Affiliations:** ^1^ State Key Laboratory of Plant Physiology and Biochemistry, College of Life Sciences, Zhejiang University, Hangzhou, Zhejiang, China; ^2^ Institute of Fundamental and Transdisciplinary, Zhejiang University, Hangzhou, Zhejiang, China; ^3^ College of Forestry and Biotechnology, Zhejiang A & F University, Hangzhou, China; ^4^ State Key Laboratory of Tree Genetics and Breeding, Chinese Academy of Forestry, Beijing, China; ^5^ Department of Forestry and Environmental Resources, North Carolina State University, Raleigh, NC, United States; ^6^ School of Environmental Science and Engineering, Tianjin University, Tianjin, China

**Keywords:** forest tree, woody plants, functional genomics, breeding, genome editing

Forest ecosystems—one of the biggest carbon sinks in the world—play a key role in terrestrial biodiversity and carbon sequestration. As important sustainable resources, trees serve as rich sources of agronomic and economic traits, providing wood, pulp and paper, fiber-related products, energy, and chemical products. Over the past few decades, conventional crossbreeding has helped generate plant varieties with improved agronomic and economic traits. However, conventional crossbreeding in forestry is time-intensive and has reached a bottleneck. Thus, improvement in growth and agronomic and economically important traits in tree species requires attention. Biotechnology has recently resulted in great progress in crop breeding, owing to the development of high-quality genome assembly and annotation tools, gene identification techniques, and efficient gene editing. Nonetheless, compared to crop species, extensive efforts are needed for the assembly and annotation of high-quality genomes, identification of key genes regulating agronomic and economically important traits, and highly efficient gene editing in tree species that exhibit high heterozygosity.

This Frontiers Research Topic aims to present the latest fundamental discoveries in the field of forest tree genomics, including genetic studies focusing on the genes and pathways associated with key agronomic and economically important traits, molecular mechanisms underlying secondary growth regulation, and the potential utilization of biotechnology in genetic improvement of woody plant species.

This volume is organized into the following sections: (1) genome assembly and annotation; (2) functional identification of key genes regulating tree growth, vascular development, and stress response; and (3) genetic transformation and gene editing in woody plants.

## Genome assembly

The first tree species with a completely sequenced genome was *Populus trichocarpa* (Tuskan et al., 2006). With the increase in sequencing depth and length and decrease in sequencing cost, the genomes of several woody plants, including poplar, have been sequenced (Liu et al., 2019; Liu et al., 2020; Huang et al., 2021; Niu et al., 2022; Zhang et al., 2024). Researchers have also been able to sequence a nearly gapless, highly contiguous genome of a doubled haploid line of *P. ussuriensis*, whose 19 chromosomes have been assembled from telomere to telomere (Liu et al., 2024; Niu and Li, 2024; Shi et al., 2024). Additionally, Sahu et al. have assembled the genome of Indian rosewood at the chromosome level, providing the foundation for studying tree growth and wood formation in this species. The assembly of this reference genome is likely to promote research on the formation of heartwood, which is an economically important wood.

## Functional identification of key genes regulating the growth, vascular development, and stress response of tree species

Tree biomass is determined by longitudinal and lateral growth. The cultivation of fast-growing trees is of great interest to produce adequate biomass in a shorter time. In this regard, researchers have attempted to identify the key genes regulating the growth rate of tree species for genetic improvement. Akutsu et al. used genomic selection (GS) for growth characteristics in an open-pollinated breeding population of Korean red pine (*Pinus densiflora*) and concluded that the trained GS model was more effective than traditional breeding methods. Kang et al. conducted GS prediction on a half-sib progeny population of *Shorea macrophylla* using six methods, and showed that GS with GWAS-based SNP selection was useful for breeding tree species.

Wood formation, or secondary growth, is a biological process specific to woody plants. The continuous activities of the cambium, comprising cambial cell proliferation, cell expansion, secondary wall thickening, and programmed cell death (PCD), produce xylem (wood) (Du et al., 2023). Key regulators of cambial cell proliferation and secondary wall thickening in poplar trees have been identified using forward and reverse genetics (Hu et al., 2022; Kim et al., 2022; Luo and Li, 2022; Dai et al., 2023; Li et al., 2024; Zhang et al., 2024; Zhou et al., 2024; Zhu and Li, 2024). However, studies on PCD in xylem cells are still limited.

Liu et al. stained different xylem cells using different dyes and sorted living cells, early PCD cells, and late PCD cells using flow cytometry. This method can also improve the analysis of gene expression dynamics along the continuous developmental stages of PCD. Guérin et al. generated dominant repressor poplar lines for *PtaPLATZ18*, which encodes an A/T-rich and zinc-binding protein. *PtaPLATZ18-SRDX* transgenic lines exhibited wider xylem compared to the wildtype plants, with a higher lignin content in transgenic wood. Moreover, the transgenic plants exhibited significantly increased height, suggesting the potential of this gene in promoting tree growth and wood production.

Tree growth is immensely affected by environmental factors such as drought and high temperatures (Jiang et al., 2023), which induce several physiological and molecular processes, such as abscisic acid synthesis in roots and leaves. Yu et al. analyzed the transcriptomic profile of *Phoebe bournei*, an important afforestation tree species in the subtropical region of China, exposed to drought stress. Through gene co-expression network analysis, they identified two core transcription factors, TGA4 and APRR2, involved in drought. Nonetheless, further genetic experiments are required to determine the functions of the candidate genes.

## Genetic transformation and gene editing in woody plants

Genetic transformation is vital to characterize the function of a gene. In addition to genome assembly, successful genetic transformation using stems (Song et al., 2006) and leaves as explants (Li et al., 2017) makes *P. trichocarpa* a model species. As *P. trichocarpa* is an endemic species, researchers have resort to other poplar species, such as *P. alba*, *P. alba* × *P. glandulosa*, and *P. tremula*. Transformation in poplar, which relies on tissue culture, is mostly mediated by *Agrobacterium tumefaciens*, although *A. rhizogenes*-mediated transformation is independent of tissue culture and produces hairy roots, from which plants can regenerate. In this regard, Ying et al. reviewed recent advances in *A. rhizogenes*-mediated transformation and Ri breeding in woody plants and emphasized its potential application in the difficult-to-propagate woody species.

Gene editing has been increasingly applied for the genetic improvement of plant species because of its high precision (Borthakur et al., 2022). Fan et al. first used the type II clustered regularly interspaced short palindromic repeats (CRISPR)-associated protein (Cas9) system in *P. tomentosa* (Fan et al., 2015). Furthermore, the CRISPR-Cas12 system has been used to knock out multiple targets of *Phytoene desaturase 8* in poplar (An et al., 2020). In this study, An et al. also evaluated the effects of temperature on gene editing efficiency and observed that the majority of editing included large-fragment deletions. Similarly, Movahedi et al. examined the effects of three factors on editing and showed that high *Agrobacteria* concentration, increased DDT number, and optimized homologous arm length resulted in efficient homology-directed repair. Currently, the CRISPR-Cas system is most commonly used for gene editing in poplar, but different woody plant species may need different CRISPR-Cas systems. Thus, increased efforts are needed to test the efficiency of gene editing in woody plants other than poplar.

This Research Topic incorporates articles on genome assembly and annotation; functional identification of key genes regulating tree growth, vascular development, and stress response; and genetic transformation and gene editing in woody plants, and serves as a valuable resource for studying forest tree breeding and functional genomics.
